# Repeat self-harm hospitalizations in Canada: a survival analysis

**DOI:** 10.1186/s40621-025-00576-y

**Published:** 2025-05-09

**Authors:** Li Liu, Gisèle Contreras, Wendy Thompson

**Affiliations:** Public Health Agency of Canada, 785 Carling Avenue, Ottawa, ON K1S 5H4 Canada

**Keywords:** Repeat hospitalization, Self-inflict injury, Suicide, Suicide attempt, Substance, Mental health

## Abstract

**Background:**

Repeat self-harm hospitalizations are associated with a greater risk of suicide and place a substantial burden on the healthcare system. In Canada, despite growing awareness of self-harm as a public heath issue, most existing research has focused on the prevalence of self-harm, with less attention given to repeat admissions. This study aims to assess the risk of repeat self-harm hospitalizations in Canada and identify population subgroups at higher risk.

**Methods:**

We included 74,055 patients discharged between April 2016 and March 2022, with self-harm hospitalizations recorded in the Canadian Institute for Health Information’s Discharge Abstract Database and the Ontario Mental Health Reporting System. After an initial self-harm hospitalization, patients were followed for repeat admissions during the study period. The risk of readmission was estimated using Kaplan-Meier survival analysis, while hazard ratios for factors such as sex, age group, method of self-harm and the presence of a mental disorder diagnosis, were calculated using Cox regression models.

**Results:**

Among patients hospitalized for self-harm, the risk of readmission was 9.3% within one year and 13.0% within three years of the index hospitalization. Three-quarters of readmissions occurred within the first year, and 90% occurred within two years. Females had a higher risk of readmission than males (hazard ratio = 1.32), with the highest risk observed among females aged 10–14 years (19.2% within three years), while patients aged 65 years and older had the lowest risk for both males and females. Females who self-harmed by cutting and patients of both sexes who used substance-related poisoning methods, as well as patients with a mental disorder diagnosis, were also at greater risk of readmissions.

**Conclusion:**

In Canada, approximately one in ten patients hospitalized for self-harm were readmitted, with most readmissions occurring within the subsequent first year. Certain subgroups, including females, young girls, individuals who engaged in self-harm through cutting or substance use, and those with a mental disorder, face higher risks. This study provides insights to guide targeted interventions aimed at preventing recurrence, informing resource allocation, and emphasizing the need for comprehensive mental health support to improve outcomes for at-risk individuals.

**Supplementary Information:**

The online version contains supplementary material available at 10.1186/s40621-025-00576-y.

## Introduction

Self-harm, including self-injury and self-poisoning, irrespective of motive or suicidal intent [1], is a serious public health issue. In 2018, approximately 25,000 people in Canada were hospitalized or died as a result of self-harm [1]. The consequences of self-harm are many, including psychosocial problems [2] and premature death from other causes [3–5]. Self-harm is one of the strongest predictors of suicide, with studies showing that the risk of suicide within 12 months of hospital discharge is 50 to 131 times higher in people who have self-harmed compared to the general population [6–10]. Finally, self-harm is also associated with significant costs to the health care system. It is estimated that, in 2018 in Canada, costs related to self-harm were approximately 305$ millions for hospitalizations, with an additional cost of 178$ millions for emergency department visits [11].

Evidence indicates that a history of self-harm is one of the strongest and consistent predictors of repeat self-harm [12]. Repeat self-harm is not uncommon, with systematic reviews and meta-analyses estimating a pooled one-year repetition rate of 16% after first time self-harm hospital presentation [13, 14], while other studies reported rates closer to 20% [15–17]. Risk of repetition increases with the number of previous presentations of self-harm [16, 17]. One study determined that the 12-month repetition rates were 14% among those with no previous presentations (i.e., first-time presenters), and increased to 37%, 50%, 62%, and 70% for those with one, two, three and four previous presentations, respectively [18]. Time between repetition also appears to be influenced by the number of episodes, with time between episodes being shorter for those with multiple episodes compared to those with fewer episodes [16]. Among persons who self-harm, those with multiple presentations of self-harm hospitalizations have more than double the risk of suicide than those with only one self-harm hospitalization [19]. Few studies have reported on rates of repeat self-harm hospitalizations in Canada, with data limited to individual provinces. A study of Ontario residents presenting to an emergency department (ED) after a first intentional overdose reported that 17% returned with a repeat overdose after a median interval of 288 days and that, among them, 5.5% had multiple repeat episodes [20]. Another study reported that 1 in 5 Ontario adults admitted to the emergency department for self-harm had a repeat visit to an ED for self-harm within 5 years of their index visit [21]. Finally, Ontario adolescents who presented to EDs with self-harm were almost 5 times more likely to have repeat ED visits or hospital admissions related to self-harm than propensity-matched controls who presented to EDs for reasons other than self-harm, over a 5-year follow-up period [22].

Mental health conditions are strongly linked to an increased risk of repeat self-harm [12]. One study found that nearly 90% of individuals with five or more ED presentations for self-harm had a history of mental or behavioral disorders [23]. Additionally, having a greater number of psychiatric disorders has been linked to a higher risk of self-harm repetition [12]. Research has also highlighted the impact of specific mental health diagnoses, identifying personality disorders as significant risk factors for repeat self-harm episodes [24, 25]. There is also evidence supporting associations between repetition and conditions such as schizophrenia and alcohol or drug abuse or dependence [12]. Other risk factors have been less studied or have demonstrated inconsistent associations. A systematic review of risk factors of repeat self-harm concluded that there was contradictory evidence on how sex affected repetition risk, with small effects observed in both directions [12]. Self-cutting as a self-harm method used in the index episode was a risk factor for repeat self-harm [18]. In another study, patients who engaged in a combination of self-poisoning and another method of self-harm in a single episode were also more likely to return to repeat self-harm compared to those who engaged in self-poisoning alone [26]. Finally, place of residence, in particular residing in more rural or remote areas, is associated with higher rates of hospitalizations [27], but little is known on its impact on repeat self-harm hospitalization. In a Canadian study, repeat self-harm hospitalizations were significantly higher in more socially deprived areas compared to less deprived areas for both males and females, while it was higher in areas with higher material deprivation but in males only [28].

Using administrative data in Canada from April 2016 to March 2022, the objectives of this study were to estimate (1) the risk of repeat self-harm hospitalizations within 30 days, 90 days, 1 year and 3 years since the index self-harm hospitalization (i.e., the first episode of self-harm hospitalization), and (2) the impacts of demographic (sex, age group, and patients’ residential remoteness) and other factors (method of self-harm and presence of a mental disorder diagnosis) on the risk of repeat self-harm hospitalization.

## Methods

### Data sources

Data on self-harm hospitalizations were obtained from the Canadian Institute for Health Information (CIHI)’s Discharge Abstract Database (DAD) and the Ontario Mental Health Reporting System (OMHRS) [29]. The DAD contains data for all discharges from publicly funded acute care facilities, including general and mental health hospitals, across all provinces and territories in Canada except Quebec. In Ontario, the province with the largest population in Canada, hospitalizations for mental health services not captured by the DAD are recorded in OMHRS. OMHRS captures 30–40% of all self-harm hospitalizations in the province [30]. For this study, OMHRS data were extracted from the Hospital Mental Health Database (HMHDB) for records prior to 2016, and from the Hospital Mental Health and Substance Use (HMHSU) database for records from 2016 onward. Data were accessed through the Public Health Agency of Canada under a data agreement with CIHI. Hospitalization data from Quebec were excluded due to limited access.

### Self-harm hospitalizations

Self-harm hospitalizations were identified using the International Statistical Classification of Diseases and Related Health Problems, 10 th Revision, Canada (ICD-10-CA), with diagnosis codes ICD-10  X60-X84, and Y87.0. In the DAD, up to 25 diagnosis codes could be recorded. A hospitalization was classified as a self-harm hospitalization if any of the diagnosis code indicated self-harm. OMHRS data do not have ICD-10 codes. Since most admissions for self-harm presented first through the emergency department (ED), CIHI developed a robust algorithm to link OMHRS data with Ontario ED data [29]. Hospital admissions recorded in OMHRS with a prior ED visit within 7 days for intentional self-harm (identifying using ICD-10 codes) were classified as self-harm hospitalizations. Data on ED visits were from CIHI’s National Ambulatory Care Reporting System (NACRS).

This study included self-harm hospitalizations among individuals aged 10 years or older who were discharged from hospitals recorded in the DAD and OMHRS between April 1st, 2016 and March 31st, 2022. The first self-harm hospitalization for each patient during the study period was designated as the index self-harm hospitalization. To minimize the impact of prior hospitalizations, a two-year “washout” period was implemented, excluding patients with hospitalizations between April 2014 and March 2016. Patients who died during their index hospitalization (1,544 patients) were also excluded. The study included index self-harm hospitalizations and any subsequent self-harm hospitalizations occurring during the study period.

### Variables of interest

We examined the relationships between repeat self-harm hospitalizations and several factors, including sex, age group, method of self-harm, the presence of mental disorders, and patients’ residential remoteness. Age groups were categorized as follows: children (10–14 years), youth (15–19 years), emerging adults (20–24 years), young adults (25–44 years), middle-aged adults (45–64 years), and older adults (65 years and over). Self-harm methods were classified into four categories: (1) poisoning by substances (ICD-10 codes X61, X62, X64 and X65), (2) poisoning by nonsubstances (X60, X63, and X66-X69), (3) cutting/piercing (X78-X79), and (4) other methods (including hanging, drowning, firearm, jumping, crashing, and other specified or unspecified means, as identified by other self-harm ICD-10 codes). Among index hospitalizations, 15.8% involved more than one self-harm method category. The majority (14.3%) of them used a combination of poisoning by substances and non-substances, and these cases were classified as poisoning by substances. For the remaining 1.5% hospitalizations involving 2–4 method categories, only the first diagnosis code was used to avoid issues with small sample sizes.

A hospitalization was classified as having a mental disorder (including mental, behavioral, and neurodevelopmental disorders) if it included an ICD-10 code in the range of F00-F99 in the DAD or was recorded in the OMHRS. The mental disorder diagnosis can encompass various types, such as the primary diagnosis, secondary diagnosis, comorbidities, or diagnosis identified pre-admission or post-admission. The remoteness of a patient’s residence was determined using the remoteness index developed by Statistics Canada [31], which considers population size and distance from service points for each census subdivision. Areas were categorized as easily accessible, accessible, less accessible, remote, and very remote [27]. All the variables referred to the characteristics of the patients’ index self-harm hospitalization at the time of admission. If a characteristic was missing for the index hospitalization, information from a follow-up hospitalization was used.

### Statistical analysis

We conducted Kaplan-Meier survival analysis to estimate the risk of repeat self-harm hospitalizations at various time points - within 30 days, 90 days, 1 year and 3 years after the index hospitalization. We used 95% confidence intervals to determine whether the risk differed significantly across groups. The analysis was stratified by sex and age group. We also calculated the percentages of repetition over time among all repeat self-harm hospitalizations. Cox regression analysis was performed to examine risk disparities across sociodemographic groups (categorized by sex, age group, and remoteness of residence) and other factors, including method of self-harm and presence of a mental disorder diagnosis. We examined log-log survival curves to ensure that the proportional hazards assumption was reasonable satisfied. Both unadjusted hazard ratios and hazard ratios adjusted accounting for the aforementioned covariates were calculated with sex stratified. For the survival analyses, only the first repeat self-harm hospitalization following the index episode was included. Finally, we examined the number and percentage of patients with multiple repeat self-harm hospitalization (1, 2, and 3 or more). SAS Enterprise Guide 7.0 software was used for the analysis.

## Results

This study analyzed 89,042 self-harm hospitalizations (including both index and follow-up) involving 74,055 patients discharged between April 1, 2016 and March 31, 2022. Of these, 46,807 patients were female, 27,186 were male, and 62 identified as a sex other than female or male. The number and percent of all and index self-harm hospitalizations are presented in Table 1. Female hospitalizations were nearly double those of male hospitalizations. Two third of the hospitalizations were related to substance use, and over three-quarters of the hospitalizations involved patients with a mental disorder diagnosis.

**Table 1:** Distribution of self-harm hospitalizations

Variable	Category	Index hospitalization		All hospitalizations	
		N	%	N	%
**Overall**		74,055	100.0	89,042	100.0
** Sex**	Female	46,807	63.2	57,824	64.9
	Male	27,186	36.7	31,131	35.0
	Other	62	0.1	87	0.1
**Age group, years**					
** Female**	10–14	4596	9.8	5541	9.6
	15–19	11,349	24.2	13,895	24.0
	20–24	6471	13.8	8568	14.8
	25–44	12,281	26.2	15,614	27.0
	45–64	9229	19.7	11,042	19.1
	65+	2881	6.2	3164	5.5
** Male**	10–14	583	2.1	650	2.1
	15–19	3381	12.4	3781	12.1
	20–24	3501	12.9	4016	12.9
	25–44	9814	36.1	11,445	36.8
	45–64	7331	27.0	8440	27.1
	65+	2576	9.5	2805	9.0
**Method**	Poisoning-nonsubstance	14,548	19.6	17,734	19.9
	Poisoning-substance related	48,958	66.1	58,562	65.8
	Cutting/piercing	6596	8.9	8132	9.1
	Other^a^	3953	5.3	4614	5.2
**Mental disorder**	Yes	57,081	77.1	68,795	77.3
	No	16,974	22.9	20,247	22.7
**Remoteness**	Easily accessible area	30,527	48.7	35,374	48.1
	Accessible area	17,010	27.2	20,313	27.6
	Less accessible area	7823	12.5	9224	12.5
	Remote area	4908	7.8	5855	8.0
	Very Remote Area	2365	3.8	2800	3.8

^a^The ‘Other’ category includes all intentional self-harm methods (ICD-10 codes X60-X84, Y87.0) other than poisoning and cutting/piercing, such as hanging, drowning, firearm, jumping, crashing, and other specified or unspecified means etc.

During the study period, the patients were followed up for 0–6 years, with a median follow-up time of 2.7 years (IQR 1.0–4.4 years), and a mean follow-up time of 2.8 years. Table 2 shows Kaplan-Meier risk estimates of repeat self-harm hospitalizations at different time points, 30 days, 90 days, 1 year, and 3 years. Overall, 3.1% (95% CI: 3.0–3.2) of patients had repeat self-harm hospitalizations within 30 days, 9.3% (95% CI: 9.1–9.6) within 1 year, and 13.0% (95% CI: 12.7–13.3) within 3 years. Females consistently exhibited a significantly higher risk of repetition compared to males. Within 1 year, 10.3% (95% CI: 10.1–10.6) of female patients were at risk of repeat self-harm hospitalizations, compared to 7.6% (95% CI: 7.3–7.9) of males; within three years, the risk was 14.5% (95% CI: 14.2–14.8) for females and 10.4% (95% CI: 10.1–10.8) for males. Across age groups, for a longer follow-up period (i.e. over one year), females ages 10–14 years tended to have the highest risk of repeat self-harm hospitalizations, while females aged 65 years or older had the lowest risk of repetition. Within 3 years, 19.2% (95% CI: 18.0–20.5) of females ages 10–14 years were at risk of repetition, more than double the risk of older females 65 years or older (8.2%, 95% CI: 7.1–9.3). However, in the short term (within 90 days follow-up), females aged 20–24 years and 25–44 years had highest risk of repetition across age groups. For males, fewer risk disparities were observed across age group compared to females, though similar to females, males aged 65 years or older had the lowest risk of repetition.

**Table 2:** Risk of repeat self-harm hospitalizations from Kaplan-Meier analysis

		30 days % (95% CI)	90 days % (95% CI)	1 year % (95% CI)	3 year % (95% CI)
**Overall**		3.1 (3.0, 3.2)	5.0 (4.9, 5.2)	9.3 (9.1, 9.6)	13.0 (12.7, 13.3)
**Sex**					
Females		3.2 (3.1, 3.4)	5.4 (5.2, 5.6)	10.3 (10.1, 10.6)	14.5 (14.2, 14.8)
Males		2.9 (2.7, 3.1)	4.4 (4.2, 4.7)	7.6 (7.3, 7.9)	10.4 (10.1, 10.8)
**Age group, years**					
** Female**					
10–14		2.6 (2.2, 3.1)	5.5 (4.9, 6.2)	12.5 (11.6, 13.5)	19.2 (18.0, 20.5)
15–19		2.9 (2.6, 3.3)	5.3 (4.9, 5.8)	10.9 (10.3, 11.5)	15.0 (14.3, 15.7)
20–24		3.9 (3.5, 4.4)	6.4 (5.8, 7.0)	11.6 (10.8, 12.4)	15.4 (14.5, 16.4)
25–44		3.8 (3.5, 4.2)	5.9 (5.5, 6.4)	10.7 (10.2, 11.3)	14.8 (14.1, 15.5)
45–64		2.9 (2.6, 3.2)	4.6 (4.1, 5.0)	8.6 (8.1, 9.2)	12.7 (11.9, 13.4)
65+		2.4 (1.9, 3.1)	3.8 (3.1, 4.6)	6.1 (5.2, 7.0)	8.2 (7.1, 9.3)
** Male**					
10–14		1.5 (0.8, 2.8)	2.8 (1.8, 4.5)	6.9 (5.0, 9.3)	11.3 (8.8, 14.5)
15–19		2.3 (1.8, 2.9)	3.6 (3.0, 4.2)	6.8 (6.0, 7.7)	9.3 (8.3, 10.4)
20–24		3.0 (2.4, 3.6)	4.7 (4.0, 5.4)	8.0 (7.1, 8.9)	11.1 (10.0, 12.3)
25–44		3.0 (2.7, 3.4)	4.5 (4.1, 5.0)	8.0 (7.5, 8.6)	11.1 (10.5, 11.8)
45–64		3.2 (2.8, 3.6)	4.9 (4.5, 5.5)	8.0 (7.4, 8.7)	10.7 (10.0, 11.5)
65+		2.8 (2.2, 3.5)	3.7 (3.0, 4.5)	5.8 (4.9, 6.8)	7.6 (6.6, 8.8)

Abbreviation: CI: confidence interval

Figure S1 (in the Supplementary material) presents the Kaplan–Meier survival plots. Steeper slopes were observed within the first year following the index hospitalization for both the overall population and subgroups, indicating a higher risk of self-harm readmission during this period. The curves tended to level off after two years, suggesting that the probability of repeat admission was low beyond that point.

Additionally, as shown in Table 3, approximately 40% of repeat hospitalizations occurred within 90 days, three-quarters within one year, and 90% within two years of the index hospitalizations for both females and males, as well as for most age groups. Exceptions were observed among younger patients aged 10–14 years and females 45–64 years, who had lower percentages of repeat hospitalizations within one year compared to other age groups.

**Table 3:** Percent of repeat self-harm hospitalizations over time among patients with repeat self-harm hospitalizations

		N	Percent, %					
			7 days	30 days	90 days	1 year	2 year	3 year
**Overall**		8894	13.5	25.7	41.4	74.0	89.5	95.8
**Sex**								
Females		6228	12.0	24.1	40.0	73.7	89.2	95.7
Males		2653	17.2	29.5	44.8	74.7	90.2	95.8
**Age group, years**								
** Female**								
10–14		777	4.5	16.2	33.3	71.6	89.3	96.5
15–19		1555	10.8	21.2	38.1	74.3	89.0	95.2
20–24		901	15.5	27.5	44.4	77.7	91.2	97.2
25–44		1681	14.7	27.8	42.7	74.5	89.1	95.8
45–64		1102	10.5	24.0	37.7	69.1	87.6	94.5
65+		212	19.3	32.5	50.5	77.8	89.6	96.7
** Male**								
10–14		61	8.2	14.8	27.9	63.9	85.2	93.4
15–19		299	15.4	26.1	40.1	74.2	88.3	95.0
20–24		358	18.4	28.8	45.0	74.6	89.9	96.6
25–44		1019	16.8	28.7	43.1	73.3	89.6	94.9
45–64		739	18.1	31.1	48.4	76.6	91.3	96.5
65+		177	18.6	40.1	53.1	79.7	94.4	98.9

Table 4 shows the hazard ratios for repeat self-harm hospitalizations based on Cox regression models. After adjusting for other variables, females had 1.32 times the risk (95% CI: 1.26–1.39) of self-harm hospitalizations compared to males. Patients aged 65 years or older had a significantly lower risk of repeat self-harm hospitalizations compared to younger patients. The highest hazard ratio (2.34, 95% CI: 2.00–2.75) was observed among girls aged 10–14 years, using females 65 years or older as reference groups, although some confidence intervals overlapped. Males younger than 65 years did not show significantly different risk of repeat hospitalizations across age group. The lowest risk of repetition was observed among patients aged 65 years or older for both females and males.

**Table 4:** Hazard ratio of repeat self-harm hospitalizations

Variable		All sexes		Female		Male	
		Hazard ratio	Adjusted hazard ratio	Hazard ratio	Adjusted hazard ratio	Hazard ratio	Adjusted hazard ratio
**Sex**							
Male		(ref)	(ref)	──	──	──	──
Female		**1.40 (1.34**,** 1.46)**	**1.32 (1.26**,** 1.39)**	──	──	──	──
**Age group, years**							
10–14		**2.31 (2.05**,** 2.61)**	**2.07 (1.82**,** 2.34)**	**2.36 (2.03**,** 2.75)**	**2.34 (2.00**,** 2.75)**	**1.47 (1.10**,** 1.97)**	**1.46 (1.09**,** 1.95)**
15–19		**1.79 (1.60**,** 2.00)**	**1.68 (1.50**,** 1.89)**	**1.92 (1.66**,** 2.21)**	**1.91 (1.65**,** 2.23)**	**1.24 (1.03**,** 1.49)**	**1.27 (1.05**,** 1.54)**
20–24		**1.81 (1.62**,** 2.03)**	**1.76 (1.56**,** 1.98)**	**1.96 (1.69**,** 2.28)**	**1.96 (1.67**,** 2.30)**	**1.47 (1.23**,** 1.77)**	**1.50 (1.24**,** 1.81)**
25–44		**1.73 (1.55**,** 1.92)**	**1.75 (1.56**,** 1.95)**	**1.89 (1.64**,** 2.18)**	**1.91 (1.65**,** 2.23)**	**1.51 (1.29**,** 1.77)**	**1.54 (1.31**,** 1.82)**
45–64		**1.54 (1.38**,** 1.71)**	**1.55 (1.38**,** 1.74)**	**1.59 (1.37**,** 1.84)**	**1.64 (1.40**,** 1.91)**	**1.45 (1.23**,** 1.70)**	**1.45 (1.23**,** 1.72)**
65+		(ref)	(ref)	(ref)	(ref)	(ref)	(ref)
**Method**							
Poisoning-nonsubstance related		(ref)	(ref)	(ref)	(ref)	(ref)	(ref)
Poisoning-substance related		**1.06 (1.01**,** 1.12)**	**1.14 (1.06**,** 1.21)**	1.05 (0.99, 1.12)	**1.13 (1.06**,** 1.21)**	**1.18 (1.05**,** 1.32)**	**1.17 (1.04**,** 1.32)**
Cutting/piercing		1.07 (0.99, 1.17)	**1.19 (1.08**,** 1.31)**	**1.39 (1.25**,** 1.54)**	**1.43 (1.27**,** 1.61)**	0.96 (0.82, 1.11)	0.92 (0.78, 1.09)
Other^a^		**0.79 (0.70**,** 0.89)**	0.92 (0.81, 1.04)	1.09 (0.93, 1.27)	1.13 (0.96, 1.33)	**0.75 (0.62**,** 0.90)**	**0.75 (0.62**,** 0.91)**
**Mental disorder**							
No		(ref)	(ref)	(ref)	(ref)	(ref)	(ref)
Yes		**1.10 (1.04**,** 1.15)**	**1.17 (1.11**,** 1.24)**	**1.12 (1.05**,** 1.19)**	**1.19 (1.11**,** 1.26)**	1.05 (0.96, 1.15)	**1.15 (1.04**,** 1.26)**
**Remoteness**							
Easily accessible area		(ref)	(ref)	(ref)	(ref)	(ref)	(ref)
Accessible area		1.04 (0.98, 1.09)	1.04 (0.98, 1.09)	**1.10 (1.03**,** 1.17)**	**1.08 (1.02**,** 1.16)**	0.91 (0.83, 1.01)	**0.91 (0.82**,** 1.00)**
Less accessible area		**1.08 (1.01**,** 1.16)**	**1.08 (1.01**,** 1.16)**	**1.10 (1.02**,** 1.20)**	1.08 (1.00, 1.18)	1.04 (0.92, 1.18)	1.03 (0.91, 1.17)
Remote area		**1.13 (1.05**,** 1.23)**	**1.13 (1.05**,** 1.23)**	**1.21 (1.10**,** 1.33)**	**1.19 (1.08**,** 1.30)**	0.92 (0.78, 1.08)	0.93 (0.79, 1.09)
Very remote area		1.06 (0.94, 1.19)	1.06 (0.94, 1.19)	1.07 (0.93, 1.22)	1.04 (0.91, 1.19)	0.98 (0.78, 1.24)	1.02 (0.81, 1.30)

Bolded values indicate statistically significant at *p* < 0.05

^a^The ‘Other’ category includes all intentional self-harm methods (ICD-10 codes X60-X84, Y87.0) other than poisoning and cutting/piercing, such as hanging, drowning, firearm, jumping, crashing, and other specified or unspecified means etc

Females who self-harmed by cutting or piercing had a significantly higher risk of repeat hospitalization compared to those self-harmed by non-substance-related poisoning (adjusted hazard ratio: 1.43, 95% CI: 1.27–1.61), but this was not observed in males. Those who used substance-related poisoning had a slightly higher risk than those using non-substance-related poisoning in both sexes (adjusted hazard ratio: 1.14, 95% CI: 1.06–1.21). A significantly higher risk was also observed among patients with a mental illness diagnosis, with an adjusted hazard ratio of 1.17 (95% CI: 1.11–1.24). Using accessible area as the reference, females in accessible, less accessible, or remote areas had a significantly higher risk of repeat self-harm, but this was not the case for females in very remote area. For males, the only significant finding was that those living in accessible area had a lower risk of repetition compared to those in easily accessible area.

Moreover, we briefly examined multiple repetitions of self-harm hospitalizations, as shown in Table 5. During the study period, 8.7% of patients with self-harm hospitalizations had only one repeat hospitalization, 1.9% had two repetitions, and 1.4% experienced three or more repeat self-harm hospitalizations. Among patients with repeat admissions, nearly three-quarters (72.6%) had only one repetition, 15.9% had two repetitions, and 11.5% had three or more repetitions. A larger proportion of females had multiple repetitions compared to males. Specifically, 12.9% of females with repeat hospitalizations experienced three or more repetitions, compared to 8.3% of males. Additionally, females aged 20–44 years had the highest percentage of multiple self-harm hospitalizations, with one-third or more having more than one repeat episodes. Among males, about a quarter of those aged 15–64 years had multiple repetitions.

**Table 5:** Percent of patients with repeat self-harm hospitalizations by number of repetitions among all self-harm patients and among patients with repeat self-harm hospitalizations

		Number of patients	% with repetition	Number of repetitions					
				1	2	3+	1	2	3+
				% among all self-harm patients			% among self-harm patients with repetition		
**Overall**		74,055	12.0	8.7	1.9	1.4	72.6	15.9	11.5
**Sex**									
** Female**		46,808	13.3	9.4	2.2	1.7	70.7	16.3	12.9
** Male**		27,183	9.8	7.5	1.4	0.8	77.2	14.7	8.2
**Age group, years**									
** Female**									
10–14		4868	16.0	11.9	2.6	1.5	74.4	16.1	9.5
15–19		11,294	13.8	9.9	2.1	1.8	71.8	15.2	13.0
20–24		6354	14.2	9.5	2.4	2.3	66.7	17.1	16.2
25–44		12,242	13.7	9.3	2.3	2.1	67.6	17.1	15.3
45–64		9193	12.0	8.7	2.0	1.3	72.7	16.9	10.4
65+		2857	7.4	6.1	1.1	0.3	81.6	14.2	4.2
** Male**									
10–14		612	10.0	8.8	─^a^	─^a^	88.5	─^a^	─^a^
15–19		3408	8.8	6.8	1.2	0.7	77.6	14.0	8.4
20–24		3485	10.3	7.9	1.5	0.8	77.4	15.1	7.5
25–44		9793	10.4	7.9	1.7	0.8	76.1	16.1	7.9
45–64		7320	10.1	7.7	1.4	1.0	75.8	14.2	10.0
65+		2565	6.9	5.8	0.8	0.3	84.2	11.3	4.5

^a^Data are suppressed due to count < 5

## Discussion

This study aimed to estimate the risk of repeat self-harm hospitalizations in Canada, identify high-risk population groups, and examine the risk factors influencing the likelihood of repetition. Our findings indicate that nearly one in ten patients experienced hospital readmissions due to self-harm within one year of their initial hospitalization, and 13% within three years. Among those with repeat self-harm hospitalizations, three-quarters occurred within the first year and 90% occurred within two years. Females, young girls, females who self-harmed using cutting/piercing or patients who self-harmed by substance-related poisoning, and patients with a mental health diagnosis were found to have higher risk of repeat self-harm hospitalizations. To our knowledge, this is the first population-based, nationwide study in Canada to estimate the risk of hospital readmission following an index self-harm hospital discharge. Most Canadian research on self-harm focuses on specific provinces or age groups, and often emphasizes hospitalization rates [22, 32]​.

Our study observed that the risk of repeat self-harm hospitalizations in Canada is lower than the 16–24% risk reported in systematic reviews and meta-analyses [33–35]. One possible explanation is that our study only included patients admitted to the hospital due to self-harm, focusing on more severe cases, whereas systematic reviews included data from a broader range of self-harm incidences. Moreover, those systematic reviews and meta-analyses included few Canadian studies, and the risk of self-harm repetition may vary significantly across countries. For example, a study from Sri Lanka reported a 12-month repetition rate of only 3.1% [36], while a study in Northern Ireland found that 41% of self-harm emergency presentations had repeat visits for self-harm within 12 months. Our findings also revealed that the incidence of self-harm hospital readmissions is highest immediately following the initial hospitalization, with the majority occurring within the first and second years. These results align with findings from most relevant studies [4, 16, 37] and highlight the critical importance of follow-up care and timely interventions and support immediately after self-harm hospitalizations.

Sex differences in repeat self-harm hospitalizations were observed in this study. Not only did females have higher rates of self-harm hospitalizations compared to males in Canada [27, 30], but this study also found that females showed a significantly higher risk of repetition throughout most of the follow-up period. Mental illness is a significant factor associated with self-harm hospitalizations [38]. In Canada, females generally report a higher prevalence of mental illness [39, 40] and worse mental health outcomes [41] compared to males, which may contribute to the higher rates of repeat self-harm hospitalization. Social pressures, relationship issues, trauma and other psychosocial stressors may disproportionately impact females, leading to self-harm as a coping mechanism for emotional distress. Females may also be more likely to seek help or to be identified by the healthcare system for support, whereas males are generally more likely to use lethal methods, resulting in higher rates of suicide deaths [42]. Moreover, due to the lack of linked mortality data, we did not exclude deaths during the follow-up period. This may have had a greater impact on males than female, given the higher suicide mortality rate in Canada for males [42], potentially offsetting the disparities in risk of repeat self-harm hospitalizations between males and females.

A higher risk of repeat self-harm hospital presentations among young girls and adolescents has been observed in other studies [16, 22, 43]. For instance, a Canadian study conducted among Ontario adolescents aged 13–17 years reported that one in three individuals with self-harm-related emergency department visits experienced repeat self-harm emergency visits or hospital admissions, and female self-harm patients were at higher risk of repetition compared to non-self-harm patients [22]. Our study observed that females aged 10–14 years had the highest risk of repeat self-harm hospital admissions over a long term (3-year) follow-up period, while females aged 20–24 years showed a relatively high risk within a shorter timeframe (90 days) after their index hospitalization. These findings suggest that self-harm may have the most prolonged impact on young girls. Mental health conditions, psychiatric disorder, substance use issues, and concussions or traumatic brain injury may contribute to the elevated risk, indicating a need for extended follow-up care for this population [22]. 

Risk factors for self-harm repetition identified in our study included the use of substance-related poisoning or cutting/piercing methods for self-harm and pre-existing mental health issues. These risk factors align with findings from other studies [12, 37, 44, 45]. The results underscore the importance of targeted interventions and prevention measures to address these specific vulnerabilities. A Canadian study reported that rates of self-harm hospitalizations increased with higher levels of rurality [27], but this pattern may not fully apply to repeat self-harm hospitalizations. In our study, females living in more remote area, but not the most remote area, tended to have a higher risk of repetition, while males did not show an elevated risk associated with increasing rurality levels. This may be due to a combination of factors, such as barriers to timely and adequate healthcare service, mental illness and chronic disease multimorbidity, help-seeking behaviors, substance use, experience of violence, use of lethal means in suicide attempts, and area deprivation [27, 28, 46, 47]. Rural people were more likely to use firearms or hanging and less likely to use poisoning methods for suicide compared to urban people [48], which may result in higher suicide mortality rather than hospital readmission. Further studies linking hospital data with mortality records could provide a more comprehensive understanding.

Moreover, among those with hospital readmissions, our study found that the majority, approximately three-quarters, had only one repeat self-harm hospitalization, though this number varied by population subgroup. This finding may indicate the potential effectiveness of initial interventions and the resilience of a significant proportion of patients following their initial episode. It also highlights the importance of hospital care during the initial hospitalization to prevent future repetition. Future research is needed to explore and understand protective factors to better inform prevention strategies and identify patients at greater risk of recurrence.

## Strengths and limitations

Our study used hospitalization data from three databases that covered the majority of Canadian population (over 76%). In addition to the DAD, our study included self-harm hospitalizations recorded in OMHRS, which accounted for 30–40% of self-harm hospitalizations in Ontario [30]. This inclusion provides more comprehensive estimates for the province, in contrast to most previous studies that relied solely on DAD data. The relatively long follow-up period (up to six years) allowed us to more robustly identify the risk of repeat self-harm hospitalizations. We performed age- and sex- stratified analyses to help understand the disparities in self-harm hospitalization recurrence. To the best of our knowledge, this is the first national level study to evaluate the risk of hospital readmission in Canada.

However, a few limitations of this study should be noted. Firstly, without linking hospitalization data with mortality data, the study only excluded the patients who died during their index hospitalization, but deaths in other places were not censored during the study period. This may have led to a slight underestimation of the risk of hospital readmission. Additionally, this could potentially offset the disparities in the risk of repeat self-harm hospitalizations between females and males, given the higher suicide mortality rate among males in Canada. Secondly, the patients in this study were linked by encrypted health card number and the province/territory that issued the health card, rather than a patient chart or unique identifier. As a result, inaccuracies in linkage may be present and may result in underestimations. After validating with the CIHI data containing patient’s unique identifier derived from patient charts, we found that the risk estimates in this study were underestimated by less than 1% point. For a small amount of hospitalizations (1.5%), we used the first self-harm ICD-10 code recorded in DAD for each hospitalization to classify the method category of self-harm, which may lead to misclassification for the hospitalizations with multiple self-harm diagnosis codes. This study covered most jurisdictions in Canada, but excluded Quebec due to data access restrictions. Moreover, due to limited demographic variables available in administration data, we were unable to investigate certain factors, such as ethnicity, socioeconomic status, marital status, and sexual orientation. Future studies could address this gap by linking administrative data with census data to better understand the impact of these factors. In addition, ICD-10 codes were used to identify self-harm cases in this paper. However, these codes do not allow us to determine whether a self-harm case involved suicide intent. Mental disorder diagnoses were based on the ICD-10 codes recorded in a hospitalization. If a mental disorder diagnosis was not documented in a hospitalization record, misclassification may have occurred. Furthermore, misclassification of self-harm case as unintentional or undetermined may also have led to an underestimation of the self-harm cases [49].

## Conclusion

We studied repeat self-harm hospitalizations in Canada and found that overall the risk of repeat self-harm hospitalizations was 9.3% within one year and 13.0% within three years after the index self-harm hospitalization. The highest incidence rates were observed during the early period following the index hospitalization, with three-quarters of repeat hospitalizations happening within the first year and about 90% within two years. Overall, female patients had a higher risk of self-harm repetition than males. The highest risk was observed in young girls aged 10–14 years, while those 65 years or older had the lowest risk. Female patients who used cutting, piercing method or patients who used substance-related poisoning method had a higher risk of repetition compared to those who self-harm using non-substance-related poisoning. Males who used the methods other than poisoning, cutting, and piercing had the lowest risk. Additionally, patients with mental disorder showed a slightly elevated risk of repeat self-harm hospitalization. This study provides insights for populations at risk, to guide targeted interventions and prevention efforts as well as the need for comprehensive mental health support.

## Supplementary Information

Supplementary Material 1

## Data Availability

Individual level data were accessed through agreements between Public Health Agency of Canada and Canadian Institution of Health Information (CIHI). Data can be requested through CIHI https://www.cihi.ca/en/access-data-and-reports/data-holdings/make-a-data-request.
