# Supplementary material for: Repeat self-harm hospitalizations in Canada: a survival analysis
Source: Inj Epidemiol. 2025 May 9;12:26. doi: 10.1186/s40621-025-00576-y (PMC12065151; doi:10.1186/s40621-025-00576-y)
Supplement: Supplementary file 1 — Supplementary Material 1 [file 40621_2025_576_MOESM1_ESM.docx]

**Supplementary material**


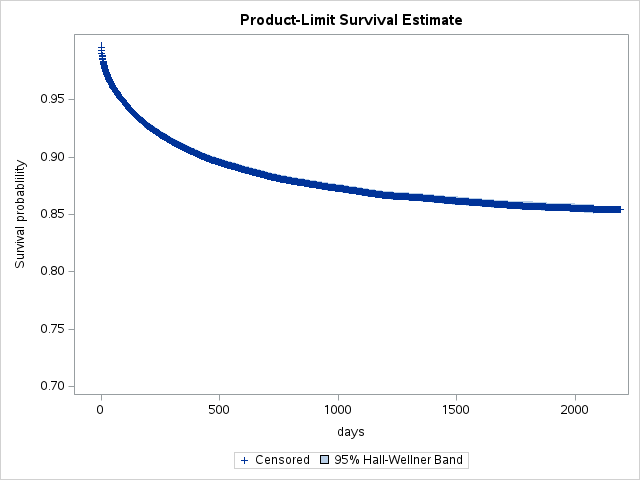


1. overall


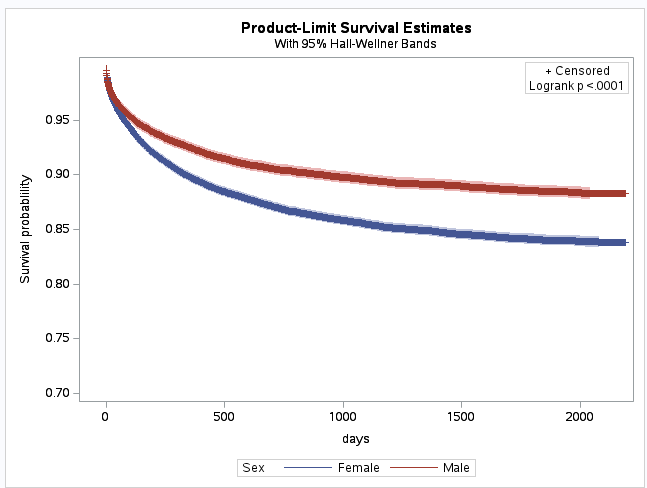


1. by sex


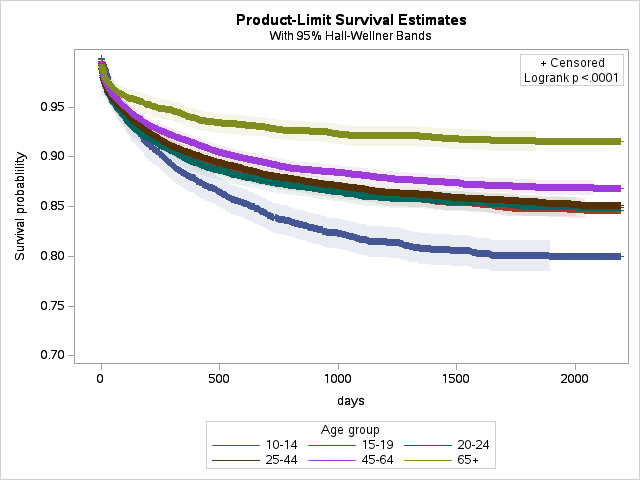


(c) by age group – female


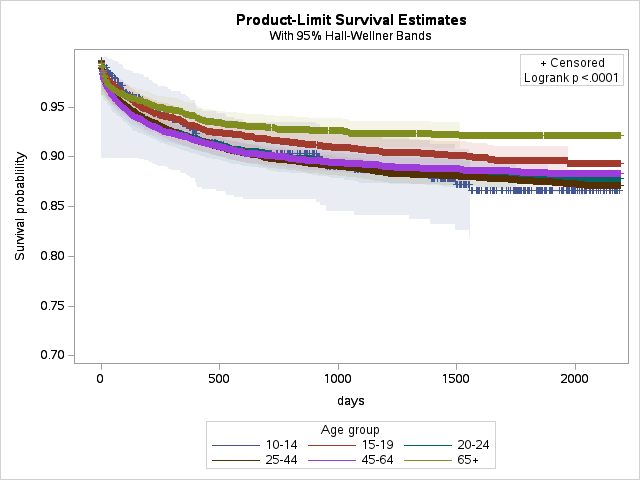


(d) by age group – male


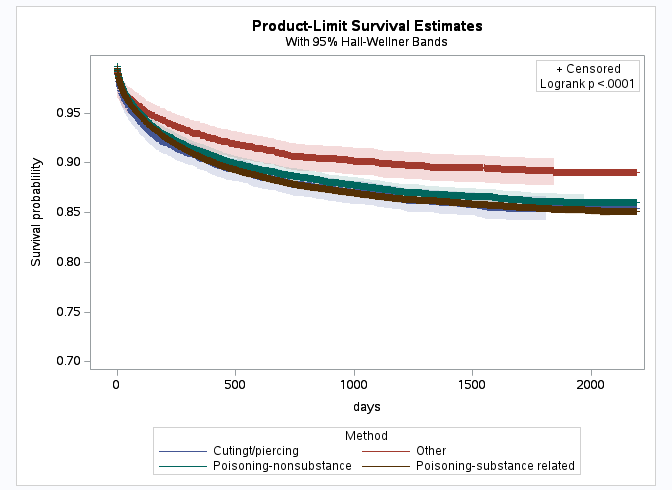


(e) by method of self-harm


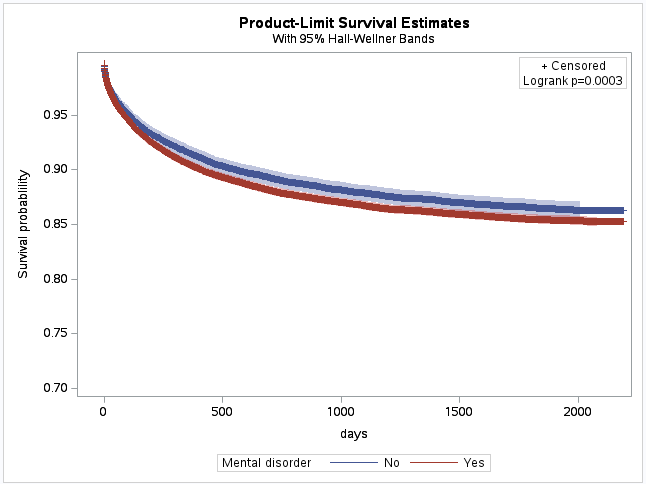


(f) by mental disorder status


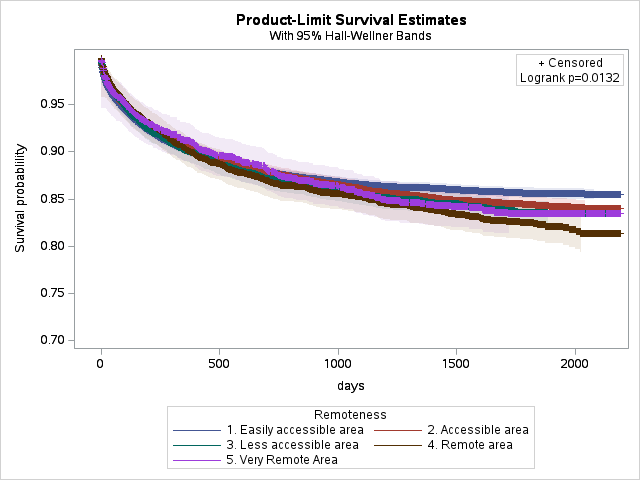


(G) by rurality of living place

Figure S1. Kaplan-Meier survival plots with log-rank test p-values for repeat self-harm hospitalizations from April 1, 2016, to March 31, 2022, overall and by sex, age group (separately for females and males), method of self-harm, mental disorder status, and rurality of residence. The log-rank test p-values < 0.05 indicate statistical differences in survival across these groups.
